# Health care needs and service use among male prison inmates in the United States: A multi-level behavioral model of prison health service utilization

**DOI:** 10.1186/s40352-017-0052-3

**Published:** 2017-06-08

**Authors:** Kathryn M. Nowotny

**Affiliations:** Department of Sociology, University of Miami, 5202 Merrick Bldg Rm 120D, Coral Gables, FL 33146 USA

**Keywords:** Health services research, Prison, Incarceration, Multilevel modeling, Health services utilization

## Abstract

**Background:**

The purpose of this study is to apply Andersen’s Behavioral Model of Health Service Use to men’s prisons to assess the direct and indirect effects of inmate predisposing characteristics through multiple types of need. Also examined are the effects of prison-specific enabling factors and the variation in use of health services across prisons. This study uses a nationally representative U.S. sample of men incarcerated in state prisons (*n* = 8816) and generalized structural equation and multilevel modeling. Five types of need—medical condition, illness, dental problem, unintentional injury, and intentional injury—are assessed for their association with use of health services.

**Results:**

Findings indicate that a number of inmate predisposing (age, race, education) and vulnerability (mood/anxiety disorder,) characteristics are associated with use of health services but are partially mediated by enabling and need factors. Each type of medical need has strong direct effects with mood/anxiety disorder emerging as the strongest total effect (including both direct effects and indirect effects through need). There is significant variation in rates of health service utilization across prisons that is not accounted for by the prison-level factors included in the multilevel model.

**Conclusions:**

The varying patterns of health service use across prisons suggest that incarceration may be an important circumstance that shapes health. In other words, where someone is incarcerated may influence their ability to access and use services in response to medical need. It is important that prisons provide integrated services for inmates with mood/anxiety disorder given high comorbidity with other health conditions.

## Background

Most states’ correctional health care spending has increased substantially due in part to the challenges of delivering health services in prisons (The Pew Charitable Trusts, 2014). Conceptually, prisons represent a “equal access” health care system in the United States (see Delgado & Humm-Delgado, 2008) that minimizes differences in economic status and health coverage similar to the Veteran’s Health Administration (Saha et al., 2008) and the Medicare program (Schneider et al, 2002). In the landmark 1976 U.S. Supreme Court case *Estelle v. Gamble*, the Court ruled that prisoners are entitled to access to care for diagnosis and treatment, a professional medical judgment, and administration of the treatment prescribed by the physician. Specifically, the Court ruled that:

Deliberate indifference to serious medical needs of prisoners constitutes the “unnecessary and wanton infliction of pain”… proscribed by the Eight Amendment. This is true whether the indifference is manifested by prison doctors in their response to the prisoner’s needs or by prison guards intentionally denying or delaying access to medical care or intentionally interfering with the treatment once prescribed. (*Estelle v. Gamble*, 429 E.S. 97, 104-05)

Thus, inmates have a constitutionally protected right to care for their “serious medical needs.”^1^ Additionally, under the Civil Rights of Institutionalized Person Act (CRIPA; 42 U.S.C. § 1997 et seq.) most jurisdictions are required to staff medical and mental health professionals to determine when an inmate is in need of medical or mental health services (jails with less than 100 detainees are exempt). However, reports indicate that nonmedical correctional staff often perform screening during intake (Patterson & Greifinger, 2006).

Traditional correctional settings are designed for punishment, incapacitation, and deterrence (Cullen & Jonson, 2011), goals which conflict with the aims of health care (Watson, Stimpson, & Hostick, 2004). Allen et al. (2010) summarize this tension stating:

Unlike most healthcare settings where the physician is only responsible for the patient’s welfare, doctors working within corrections often find themselves caught between the punitive aspect of the institutions’ mission and the best interests of their patients. This dual loyalty conflict is made that much harder by the fact that many features of the correctional system can directly conflict with optimal treatment for a patient’s medical conditions. This can include the deleterious effect of incarceration itself, especially for the mentally ill.

The healthcare infrastructure within correctional facilities can create barriers limiting access to medical care (Magee, Hult, & McMillan, 2005). Mandatory requirement of co-pays, hygiene issues, administration of wrong medications, medications stopped by mistake, delay in obtaining needed medications, allergic reactions to medications, and other errors on the part of the facility all contribute negatively to the health of inmates (Hatton, Kleffel, & Fisher, 2006). Providing diagnosis and treatment, and coordinating for transitions in care upon release from prison can be extremely beneficial for community public health (Binswanger, Redmond, Steiner, & Hicks, 2011) and in reducing recidivism (Baillargeon, Binswanger, Penn, Williams, & Murray, 2009).

The high rates of disease and illness in prisons (Binswanger, Krueger, & Steiner, 2009; Wilper et al., 2009), coupled with large incarceration rates for men and race/ethnic minority groups, suggest a need to examine the role of the prison in the administration of healthcare in U.S. society given that prisons disproportionately house individuals with disadvantaged health profiles. It has been documented that criminal-justice involved persons receive only episodic care from correctional facilities and emergency rooms (Boutwell & Freedman, 2014). The prison population, specifically, makes up 1% of the total U.S. population (PEW Center, 2008), but 11.4% of all black men aged 20 to 34, and 37.2% of black men aged 20 to 34 with less than a high school education (Pettit, 2012). Black men also have the highest mortality rates (Centers for Disease Control and Prevention, 2016) and among the lowest rates of health coverage (James, Thomas, Lillie-Blanton, & Garfield, 2007; Smedley, Stith, & Nelsen, 2003).

**Fig. 1:**
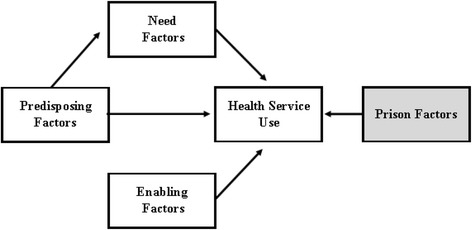
Analytical model of inmate health service use

Behavioral models of service use are useful in identifying predictors of medical care and to assess whether health service use is equitably distributed. In this study, Andersen’s Behavioral Model of Health Service Use, first developed in 1968 (Andersen, 1995), is adapted for use in prisons. Andersen’s model has been mainly used for explaining health care utilization patterns by the general population and suggests that use of health services is a function of predisposition to use services, factors that enable or impede use, and need for care, thus providing a way to conceptualize variations in utilization. The model specifies both individual and contextual determinants of health service use including individual’s predisposing characteristics (e.g., demographic variables, socioeconomic status), enabling resources (e.g., health coverage, income), and perceived need (on the part of the individual) and evaluated need (on the part of a professional) (see Fig. 1). Enabling factors are social factors that are thought to play a role in access to care. As previously discussed, within a prison access to care is supposedly the same for all inmates. Therefore, this study examines factors that shape the day-to-day experience for inmates such as offender status and hours spent in work assignment. Perceived need for care should account for the majority of the explained variability in health service use. Put another way, if service use is equitably distributed within and between prisons, individuals who are most at need will be most likely to use services, and other observed factors will not influence use of services.

Andersen’s model has been updated and modified several times by Andersen and others (Andersen, 1995; Andersen & Newman, 1973; Stein, Andersen, & Gelberg, 2007). For example, Gelberg, Andersen, and Leake (2000) developed the Behavioral Model for Vulnerable Populations. This expanded model includes specific vulnerabilities found among homeless people such as mental illness, substance use, and competing needs for health services. Notably, Leukefeld et al. (1998) propose, and later test (Garrity, Hiller, Staton, Webster, & Leukefeld, 2002; Webster et al., 2005), a version of Andersen’s model for examining use of medical services among drug-abusing offenders. They demonstrate that pre-prison demographic and drug abuse history are important predictors of medical service use.

This study is among the first to apply Andersen’s model to a diverse group of prison inmates using a multilevel and structural equation modeling framework to assess health service use across multiple need factors. Specifically examined is how varying prison contexts and inmate prison experience influence men’s’ use of health services while incarcerated, above and beyond need. Also examined are the potential indirect pathways on use of health services through variation in need by demographic and vulnerable factors that inmates bring with them—or “import” (Irwin & Cressey, 1962)—when they are incarcerated. The overall research question is: what are the factors associated with health service utilization in men’s prisons, and do utilization rates vary by prison?

## Methods

### Data

The 2004 Bureau of Justice Statistics (BJS) Survey of Inmates in State Correctional Facilities (SISCF) provides a nationally-representative sample of persons incarcerated in state prisons (United States Department of Justice. Bureau of Justice Statistics, 2007). The sample design employed a stratified, two-stage selection process. There were 1401 male facilities included in the sampling frame with the 14 largest male prisons selected with certainty. The remaining 1387 male prisons were stratified by U.S. region and population size with 222 additional facilities selected for participation. Prisoners were then randomly selected for participation within each facility. A total of 13,098 men were selected for participation with a final overall sample size of 14,499 male and female inmates. The survey asks respondents about their incarceration history, offense characteristics, family and background characteristics, drug and alcohol use and abuse, prison activities, and self-reported health, mental health, and treatment history.

The sample for the current analysis includes only men who are currently serving time who are aged 18 or older. Women are excluded since, by definition, they are housed in separate facilities. Because of skip patterns in the survey instrument, only those inmates who reported any health problems (defined below) are included. Respondents were only asked about using health services if they reported a health problem. Therefore, there is not information on people who used health services without also self-reporting a health problem. Patterns of missingness were examined and less than 5% of cases had no missing information. Therefore, a listwise deletion was performed to exclude cases with missing information. The final sample size that is used for all of the analyses in this study is 8816 men.^2^ All estimates are adjusted for the complex study design. ^3^

### Measures

*Individual-level need and service use*. The bivariate outcome included use of any medical care^4^ as a result of any of the need factors listed below. Overall, 83.6% of inmates reporting a health need used health services. “Serious medical needs” in this study were defined as reporting a current medical condition^5^, an illness since incarceration, a dental problem since incarceration, an unintentional injury since incarceration, and an intentional injury (e.g., assault) since incarceration. Almost one-third of inmates reported a medical condition (30.8%) while three-quarters reported an illness while incarcerated (73.5%). Sixty percent reported having a dental problem during their current incarceration episode (60.8%). Finally, 27.3% experienced an unintentional injury while 19.7% experienced an intentional injury. Between 13.9% and 46.0% of those in need did not use health services: 84.2% medical problem, 54.0% illness, 86.1% dental problem, 79.7% unintentional injury, 70.6% intentional injury.

*Individual-level predisposing factors.* The characteristics for all study variables are presented in Table 1. Seven inmate-level predisposing factors were included: age, race/ethnicity (non-Hispanic white, non-Hispanic black, Latino, other race), nativity (foreign born), marital status (never married), education (at least high school), employed prior to incarceration, and veteran status. The average age for this sample was 36 years. The majority of inmates were men of color with 41% black, 17% Latino, and 6% other race. Seven percent were foreign born. About half of inmates had never been married (56%) while about 70 % had a high school education (68%) and or were employed prior to incarceration (72%). Twelve percent reported serving in the military.

**Table 1:** Characteristics of study measures (*n* = 8816)

	Mean	Std. Dev.	Min	Max
Predisposing factors				
Age	35.89	10.79	18	84
Black	0.41	0.49	0	1
Hispanic	0.17	0.38	0	1
Other race	0.06	0.23	0	1
Foreign born	0.08	0.26	0	1
Never married	0.56	0.50	0	1
High school education	0.68	0.47	0	1
Prior employment	0.72	0.45	0	1
Veteran	0.12	0.32	0	1
Vulnerability factors				
Past incarceration episodes	1.09	1.21	0	3
Mood/anxiety disorder	0.23	0.42	0	1
Schizophrenia	0.04	0.39	0	1
Alcohol/drug dependence	0.42	0.49	0	1
Prison-level predisposing factors				
Proportion age 25 or younger	0.21	0.13	0	0.89
Proportion black	0.41	0.20	0	0.89
Proportion hispanic	0.18	0.16	0	0.67
Proportion violent offenders	0.51	0.20	0	0.90
Enabling factors				
Visits from family/friends				
1 in past month	0.13	0.34	0	1
2+ in past month	0.17	0.37	0	1
Phone Calls from family/friends				
1 in past week	0.17	0.37	0	1
2+ in past week	0.29	0.45	0	1
Drug offender	0.17	0.37	0	1
Violent offender	0.53	0.50	0	1
Hours in work assignment	13.87	15.7	0	40
Years incarcerated	5.45	5.65	0	44

The means are estimated using adjustments for the complex survey design

*Individual-level vulnerability factors.* There were four vulnerability predisposing factors included: previous incarceration episodes (zero, one, two, three or more; mean = 1.09), mood/anxiety disorder (i.e., depression, bipolar, anxiety, and or PTSD; 23%), schizophrenia (4%), and alcohol/drug dependence (42%).

*Contextual-level predisposing factors.* There were four prison-level predisposing factors computed by aggregating self-reported measures to the prison level, similar to previous prison studies (e.g., Jiang & Winfree, 2006; Steiner & Wooldredge, 2008). These factors included proportion of inmates aged 25 or younger, proportion of black inmates, proportion of Hispanic inmates, and proportion violent offenders. Table 1 shows that there was wide variation in proportions across prisons. For example, the mean proportion for black inmates was 0.41 with a range from 0.00 to 0.89 of inmates within a prison.

*Individual-level enabling factors*. The six inmate-level enabling factors were the number of years served to date during the current incarceration episode (continuous), offender status (violent offender and drug offender compared to other), receiving visits from family and friends in the past 12 months (0 visits, 1 visit, 2+ visits), receiving a phone call from family/friends in the past week (0 calls, 1 call, 2+ calls), and the number of hours spent in work assignment during the past week (continuous). Thirteen percent of inmates had one visit with family/friends in the past month with 17% having two or more visits. A higher percentage of inmates had phone calls with family/friends in the past week: 17% once and 29% two or more. Consistent with administrative data, 17% of inmates were drug offenders and 53% were violent offenders. Inmates spent an average of 14 h in work assignment during the past week with a range from 0 h to 40 h. Finally, the average length of time served to date was 5.45 years with a range from less than one year to 44 years.

*Analysis.*

The analysis was carried out in two parts. First, following Goodwin and Andersen (2002), stepwise logistic regression models computed adjusted odds ratios and identified the inmate predisposing, enabling, and need correlates of using health services. This analysis did not explicitly model variation in prison context or adjust for the clustering of individuals into prison, but the models are representative of the overall state prison population of men. Model 1 included the predisposing factors and vulnerability factors. Next, the enabling factors were added, and then the need factors were added to assess any potential mediating effects. If only the need factors were significant in the final model, this would provide evidence that use of services is equitably distributed for male inmates.

Second, a generalized structural equation model (GSEM) was estimated in Stata 13. Structural equation modeling (SEM) is a general modeling framework that can incorporate many common statistical methods including regression, factor analysis, and simultaneous equations, among others. This approach allows for the assessment of mediation effects (indirect pathways) that are estimated and tested in a single step that is more statistically powerful than using a multistep method. GSEM also allows for the inclusion of latent variables indicating random effects in multilevel modeling. For more information including the limitations of GSEM in Stata see http://www.stata.com/manuals13/semgsem.pdf. This analysis built off the previous analysis to test the analytical model presented in Figure 1. The multilevel logistic regression model tested the direct effects of inmate predisposing, enabling, and need factors on health service use and the indirect effects of inmate predisposing factors on health service use through need. This model also specified the prison-level predisposing factors that influence health service use by inmates, and whether use of health services varies across prisons. Indirect and total effects were decomposed using the non-linear combination command (nlcom) which provided confidence intervals for the estimates.

## Results

Table 2 presents the stepwise logistic regression models. Model 1 included only the predisposing and vulnerability factors and indicates that age (OR = 1.05, *p ≤* 0.001), black race (OR = 1.35, *p ≤* 0.001), never married (OR = 1.19, *p ≤* 0.05), having a mood and or anxiety disorder (OR = 1.35, *p ≤* 0.001), and schizophrenia (OR = 1.79, *p ≤* 0.01) are associated with use of health services. When the enabling factors were added to the model (Model 2), there were some mediating effects among the predisposing factors. Being a violent offender increased the odds of using services (OR = 1.28, *p* ≤ 0.001). Hours in work assignment had a minimal effect (OR = 1.00, *p* ≤ 0.05) while every year incarcerated contributed to 21% higher odds of using health services (OR = 1.21, *p* ≤ 0.001), but this effect lessens over time (OR = 0.99, *p* ≤ 0.001).

**Table 2:** Stepwise logistic regression models (*n* = 8816)

	Model 1				Model 2				Model 3			
	OR	*p*	CI-	CI+	OR	*p*	CI-	CI+	OR	*p*	CI-	CI+
Predisposing factors												
Age centered on 18	1.05	***	1.04	1.06	1.03	***	1.03	1.04	1.03	***	1.02	1.04
Black	1.35	***	1.17	1.56	1.27	***	1.09	1.47	1.35	***	1.14	1.59
Hispanic	0.93		0.78	1.12	0.92		0.76	1.10	0.94		0.77	1.17
Other race	1.00		0.78	1.29	1.00		0.77	1.29	0.90		0.65	1.22
Foreign born	1.20		0.93	1.53	1.26		0.98	1.62	1.36	*	1.03	1.79
Never married	1.19	*	1.04	1.36	1.10		0.96	1.27	1.14		0.98	1.33
High school education	0.99		0.87	1.13	0.88	*	0.77	1.00	0.87		0.75	1.01
Prior employment	0.95		0.84	1.09	1.00		0.87	1.14	0.96		0.82	1.12
Veteran	0.87		0.70	1.08	0.91		0.74	1.13	0.84		0.67	1.07
Vulnerability factors												
Past incarceration episodes	0.96		0.91	1.01	1.03		0.98	1.08	1.01		0.95	1.07
Mood/anxiety disorder	1.35	***	1.15	1.57	1.37	***	1.17	1.61	0.95		0.79	1.14
Schizophrenia	1.79	**	1.20	2.67	1.75	**	1.17	2.62	2.07	***	1.32	3.22
Alcohol/drug dependence	0.99		0.88	1.12	1.04		0.92	1.18	0.99		0.86	1.14
Enabling factors												
Visits from family/friends												
1 in past month					1.20	*	1.00	1.44	1.16		0.95	1.42
2+ in past month					1.19	*	1.01	1.41	1.18		0.97	1.43
Phone calls from family/friends												
1 in past week					0.96		0.81	1.13	1.04		0.86	1.25
2+ in past week					1.04		0.90	1.20	1.12		0.96	1.32
Drug offender					0.95		0.81	1.12	0.98		0.82	1.19
Violent offender					1.28	***	1.11	1.47	1.18	*	1.01	1.38
Hours in work assignment					1.00	*	1.00	1.01	1.01	*	1.00	1.01
Years incarcerated					1.21	***	1.18	1.25	1.14	***	1.10	1.18
Years incarcerated squared					0.99	***	0.99	1.00	1.00	***	0.99	1.00
Need factors												
Medical problem									7.28	***	5.81	9.12
Illness									1.88	***	1.59	2.21
Dental problem									8.22	***	7.00	9.65
Unintentional injury									6.40	***	5.10	8.03
Intentional injury									3.38	***	2.64	4.33
Model fit statistics												
Log likelihood ratio	−3778.5				−3619.5				-2873.1			
AIC	7585.1				7285.0				5802.3			
BIC	7684.2				7448.0				6000.7			

Models adjusted for complex survey design. *p ≤ 0.05; **p ≤ 0.10; ***p ≤ 0.001

The final model, Model 3, had the best overall model fit as indicated by the log likelihood ratio, AIC, and BIC. Similar to Model 2, there were some mediating effects when the need factors are included. This suggested a more complex relationship among these domains of factors than could be evaluated in a regression framework and provided empirical justification for use of a structural equation modeling framework. As expected, each of the five need factors were highly significant when controlling for predisposing and enabling factors. Having a medical condition increased the odds of using health services by a factor of 7.28 (*p* ≤ 0.001). Illness had the smallest effect with an odds ratio of 1.88 (*p* ≤ 0.001) while reporting a dental problem had the largest effect with an odds ratio of 8.22 (*p* ≤ 0.001). With regards to injury, men reporting an unintentional injury had higher odds of using health services (OR = 6.40, *p* ≤ 0.001) compared to intentional injury (OR = 3.38, *p* ≤ 0.001).

Table 3 presents the results of the generalized structural equation model. A baseline model indicated that a multilevel approach was a better fit over a single level model (*p* ≤ 0.001) and that 11.9% of the variation in health service use was due to variation between prisons (not shown). Similar to the above analysis, all five need factors were statistically significant and once need is controlled few predisposing factors remained significant predictors of health service use. Use of medical care increased with age (OR = 1.03, *p* ≤ 0.001) after controlling for need. Black men had higher odds of reporting using health services compared to white men (OR = 1.24, *p* ≤ 0.05). Finally, men who reported being diagnosed with schizophrenia had higher odds of reporting use of health services independent of need (OR = 2.36, *p* ≤ 0.001).

**Table 3:** Generalized structural equation model (*n* = 8816)

	Outcome				Need Factors																				
	Health services				Medical problem				Illness				Dental problem				Unintentional injury					Intentional injury			
	OR	*p*	CI-	CI+	OR	*p*	CI-	CI+	OR	*p*	CI-	CI+	OR	*p*	CI-	CI+	OR	*p*	CI-	CI+		OR	*p*	CI-	CI+
← Need factors																									
Medical condition	8.11	***	6.49	10.14					0.65	***	0.59	0.72	0.68	***	0.61	0.75	0.96		0.86	1.07		0.98		0.87	1.10
Illness	1.91	***	1.60	2.28	0.65	***	0.59	0.73					0.75	***	0.67	0.84	1.16	*	1.03	1.30		1.26	***	1.11	1.44
Dental problem	8.79	***	7.31	10.56	0.68	***	0.62	0.76	0.75	***	0.67	0.84					1.06		0.96	1.18		1.17	**	1.04	1.32
Unintentional injury	6.50	***	5.07	8.32	0.96		0.86	1.08	1.16	*	1.03	1.30	1.06		0.96	1.18						1.55	***	1.38	1.75
Intentional injury	3.30	***	2.56	4.27	0.99		0.88	1.12	1.27	***	1.11	1.45	1.17	**	1.04	1.32	1.55	***	1.38	1.75					
← Predisposing factors																									
Age centered on 18	1.03	***	1.02	1.04	1.05	***	1.05	1.06	1.02	***	1.01	1.02	1.03	***	1.02	1.04	0.99	***	0.98	0.99		0.99	**	0.99	1.00
Black	1.24	*	1.05	1.47	1.41	***	1.24	1.60	0.94		0.82	1.06	0.98		0.87	1.10	1.12		0.99	1.26		0.80	**	0.69	0.93
Hispanic	0.90		0.72	1.14	0.99		0.84	1.15	1.09		0.91	1.31	0.85	*	0.74	0.97	1.01		0.86	1.17		1.09		0.91	1.32
Other race	0.93		0.68	1.28	1.32	**	1.08	1.62	0.90		0.72	1.14	1.02		0.85	1.24	1.36	**	1.10	1.68		0.99		0.79	1.24
Foreign born	1.35		0.99	1.84	0.87		0.72	1.05	0.86		0.69	1.07	1.13		0.93	1.37	0.92		0.74	1.13		0.87		0.69	1.12
Never married	1.11		0.95	1.29	0.82	***	0.74	0.90	1.15	**	1.02	1.29	1.06		0.95	1.18	1.01		0.91	1.13		1.45	***	1.27	1.65
High school education	0.91		0.77	1.06	0.89	*	0.80	0.99	1.39	***	1.24	1.55	1.12	*	1.01	1.23	1.42	***	1.28	1.58		1.09		0.96	1.23
Prior employment	0.96		0.84	1.11	0.89	*	0.80	0.98	0.98		0.87	1.11	1.09		0.99	1.21	1.10		0.99	1.23		0.82	***	0.73	0.92
Veteran	0.81		0.63	1.04	0.97		0.83	1.14	1.06		0.89	1.26	0.96		0.82	1.12	1.16		1.00	1.35		1.03		0.84	1.27
← Vulnerability factors																									
Past incarceration episodes	1.01		0.95	1.08	0.97		0.93	1.02	0.98		0.94	1.03	1.03		0.99	1.06	0.98		0.94	1.02		0.97		0.93	1.02
Mood/Anxiety disorder	0.94		0.77	1.14	1.99	***	1.76	2.25	1.04		0.91	1.19	1.24	***	1.09	1.41	1.18	*	1.04	1.34		1.63	***	1.42	1.88
Schizophrenia	2.36	***	1.47	3.78	1.02		0.80	1.30	0.96		0.75	1.22	1.07		0.82	1.39	0.85		0.64	1.12		1.10		0.86	1.41
Alcohol/Drug dependence	1.01		0.88	1.16	1.01		0.91	1.12	1.15	**	1.04	1.28	1.07		0.97	1.18	0.91		0.82	1.00		1.13	*	1.02	1.26
← Enabling factors																									
Visits from family/friends																									
1 in past month	1.16		0.93	1.45																					
2+ in past month	1.19		0.97	1.44																					
Phone Calls from family/friends																									
1 in past week	1.09		0.90	1.31																					
2+ in past week	1.22	*	1.03	1.45																					
Drug offender	0.92		0.76	1.11																					
Violent offender	1.07		0.89	1.28																					
Hours in work assignment	1.00		1.00	1.01																					
Years incarcerated	1.13	***	1.09	1.18																					
Years incarcerated squared	1.00	***	0.99	1.00																					
← Prison-level predisposing factors																									
Proportion age 25 or younger	0.93		0.37	2.32																					
Proportion black	2.22	**	1.24	3.95																					
Proportion hispanic	1.84		0.95	3.56																					
Proportion violent offenders	2.03	**	1.21	3.42																					
Random effects (se)	0.23		0.14	0.38																					

Model adjusted for complex survey design *p ≤ 0.05; **p ≤ 0.10; ***p ≤ 0.001

Some predisposing factors appeared to have indirect effects on medical care by changing the need for services. However, there were no predisposing factors that consistently affected need across all five domains. Age had a positive effect on having a current medical problem (OR = 1.05, *p* ≤ 0.001), reporting an illness while incarcerated (OR = 1.02, *p* ≤ 0.001), and reporting a dental problem (OR = 1.03, *p* ≤ 0.001), but age had a small negative effect on injury, both unintentional (OR = 0.99, *p* ≤ 0.001) and intentional (OR = 0.99, *p* ≤ 0.01). Men with a high school education had lower odds of reporting a current medical problem (OR = 0.89, *p* ≤ 0.05), but had higher odds of reporting an illness (OR = 1.39, *p* ≤ 0.001), dental problem (OR = 1.12, *p* ≤ 0.05), and unintentional injury (OR = 1.42, *p* ≤ 0.001). The predisposing vulnerability factors also appeared to influence medical need. Having a mood/anxiety disorder increased the odds of having a current medical condition two-fold (OR 1.99, *p* ≤ 0.001) as well as increased the odds of having a dental problem (OR = 1.24, *p* ≤ 0.001), unintentional injury (OR = 1.18, *p* ≤ 0.05), and intentional injury (1.63, *p* ≤ 0.001). The only need factor not associated with mood/anxiety disorder was illness. Men with a history of alcohol/drug dependence had higher odds of reporting a dental problem (OR = 1.15, *p* ≤ 0.01) and suffering an intentional injury (OR = 1.13, *p* ≤ 0.05). The comorbidity of health conditions was examined further (Table 4). Mood/anxiety disorder was comorbid with all of conditions except for illness. Overall, mood/anxiety disorder and schizophrenia had the strongest association among the health conditions in this study. The patterns of co-occurrence among the physical, mental and behavioral health problems were generally maintained in the multivariate models in Table 3. Among the mental and behavioral health vulnerability factors, mood/anxiety disorder emerged an important co-occurring condition for the medical outcomes.

**Table 4:** Unadjusted pairwise comorbidity for health conditions (*n* = 8816)

		1		2		3		4		5		6		7		8
1	Medical condition															
2	Illness	0.72	***													
		(0.65, 0.79)														
3	Dental problem	0.83	***	0.79	***											
		(0.75, 0.91)		(0.72, 0.88)												
4	Unintentional injury	0.89	*	1.18	**	1.07										
		(0.80, 0.99)		(1.05, 1.31)		(0.97, 1.18)										
5	Intentional injury	0.90		1.29	***	1.15	**	1.64	***							
		(0.80, 1.02)		(1.14, 1.46)		(1.03, 1.28)		(1.46, 1.84)								
6	Mood/anxiety disorder	1.78	***	1.02		1.23	***	1.19	***	1.76	***					
		(1.60, 1.97)		(0.91, 1.15)		(1.10, 1.36)		(1.07, 1.34)		(1.56, 1.98)						
7	Schizophrenia	1.6	***	0.92		1.2		0.95		1.56	***	23.2	***			
		(1.29, 1.97)		(0.73, 1.17)		(0.96, 1.50)		(0.75, 1.20)		1.23, 1.98)		(17.4, 30.9)				
8	Alcohol/drug dependence	0.99		1.16	**	1.08		0.93		1.25	***	1.89	***	2.01	***	
		(0.90, 1.08)		(1.05, 1.28)		(0.99, 1.18)		(0.84, 1.02)		(1.12, 1.39)		(1.70, 2.09)		(1.63, 2.48)		

Odds ratios and 95% confidence intervals. Adjusted for complex sampling design. *p ≤ 0.05; **p ≤ 0.10; ***p ≤ 0.001

**Fig. 2:**
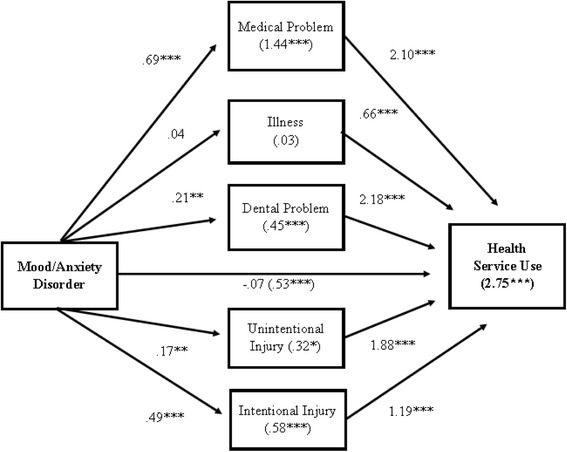
Log odds of mood/anxiety disorder predicting use of health services including direct, indirect, and total effects. Estimates derived from model in Table 3. *p ≤ 0.05; **p ≤ 0.10; ***p ≤ 0.001

Given the potentially strong indirect effects of mood/anxiety disorder, the effects on medical care were decomposed (Figure 2). The log odds of mood/anxiety disorder on any health service use was 0.53 (*p* ≤ 0.001). Medical need fully mediated this relationship so that the log odds were reduced to −0.07 (n.s.). The indirect effects of mood/anxiety disorder through the need factors were calculated as the product of the two direct effects involved in the mediation. For example, the log odds of mood/anxiety disorder on having a medical problem was 0.69 and the log odds of having a medical problem on any health service use was 2.10 for an indirect effect of 1.44 log odds or a 4.22 odds ratio. The total effect of mood/anxiety disorder on any health treatment was calculated by combining the direct effect and the five mediation (indirect) effects where the total effect was the sum of log odds of the direct and indirect effects. The total effect of mood/anxiety disorder is 2.75 (OR = 15.7, *p* ≤ 0.001) which was substantially larger than the direct effect (OR = 0.93). This means that given variation in medical problems among inmates with mood/anxiety disorder, these inmates had 15 times the odds of using medical services while incarcerated compared to inmates without mood/anxiety disorder.

Similar to Model 3 in Table 2, the only enabling factors that were significant were years incarcerated (OR = 1.13, *p* ≤ 0.001), years incarcerated squared (OR = 1.00, *p* ≤ 0.001), and having two or more phone calls from family/friends in the past week (OR = 1.22, *p* ≤ 0.05). Finally, two prison-level predisposing factors affected use of medical care including proportion black men (OR = 2.22, *p* ≤ 0.01) and proportion violent offenders (OR = 2.03, *p* ≤ 0.01). The random effects were significant (0.23, 95% CI = 0.14, 0.38) indicating variation in use of health services across prisons that was not explained by the prison-level predisposing factors examined here. The random effects were reduced from 0.45 in the baseline model (not shown) after inmate- and prison-level factors were accounted for.

## Discussion

Using an adapted version of Andersen’s Behavioral Model of Health Service Use, this study found that between 13.9% and 46.0% of men with medical health needs did not use health services in prison. Men were most likely to use health services in response to a medical condition or dental problem, and were least likely to use health services for an illness. Men are also less likely to seek medical care for an intentional injury compared to unintentional injury. This may be due to the seriousness of the injuries. It is also possible that inmates are hesitant to use health services for intentional injuries for fear of getting in trouble with correctional staff. There are a number of findings related to prison-level factors and inmate-level predisposing and enabling factors that warrant further discussion.

First, inmates in the United States have a constitutionally protected right to health care. Therefore, we should expect uniform patterns of utilization across prisons after accounting for need. However, there is significant variation between prisons in rates of health service use ranging from 50 to 94% (95% interval) of inmates in need using health services. This indicates that, not even accounting for the quality of services, not all inmates are having their service needs met despite constitutional protections. In other words, the multilevel model shows unexplained variation in utilization rates between prisons (after controlling for inmate-level differences) that are unexpected given legal mandates in the Unites States. For example, the proportion of violent offenders is positively associated with rates of medical care utilization. This could indicate differences in availability of health services based on security level since violent offenders tend to be incarcerated in higher security prisons. It was also found that the proportion of black men positively influence use of health services. This may be due to regional or state-level variation since black adults are clustered in specific geographic areas which shape inmate composition (Mauer & King, 2007; Sakala, 2014). These findings suggest that (1) where someone is incarcerated may influence their ability to access and use health services; and (2) further analysis is needed to better examine the prison context including consideration of objective prison enabling factors such as staffing, size of prison, and overcrowding. This is especially important given recent court orders to improve correctional health care delivery within several states (e.g., California, Arizona). Nevertheless, the findings emphasize that, overall, health service utilization in prison cannot be fully explained by inmate characteristics.

Second, findings indicate that inequalities in use of services while incarcerated seem to be largely driven by differences in predisposing factors on need. Although this is a promising finding, this particular analysis addresses differences in health services use regardless of why inmates may have differential levels of need. More specifically, research on health service use that takes into account health selection effects is greatly needed. For example, after adjusting for differences in need, black men are significantly more likely to utilize treatment compared to white men. These findings suggest that prisons may be an important site for healthcare for black men in U.S. society given their low levels of access to healthcare outside of prison and their high rates of incarceration (see also (Nowotny & Kathryn, 2015)). Research has consistently documented black-white disparities in healthcare for noninstitutionalized adults (Hayward, Miles, Crimmins, & Yang, 2000; Marmot, 2005). For example, a 2007 Kaiser Family Foundation report provides evidence for racial differences in health insurance coverage, access to primary care, and treatment for specific medical conditions (James et al., 2007). Some studies find that racial disparities persist even after adjustment for socioeconomic differences and other healthcare-related factors (Kressin & Petersen, 2001; Mayberry, Mili, & Ofili, 2000). It may be that the reversal of the disparity in prisons is due to black men’s motivation to seek care given their limited ability to access care in the community.

Among the vulnerability factors, inmates with mood/anxiety disorder are more likely to have medical need. In total, inmates with mood/anxiety disorder are 15 times more likely to use health services in prison. It is especially concerning that inmates with mood/anxiety disorder have higher odds of experiencing both unintentional and intentional injuries while incarcerated. This speaks to the vulnerability of inmates with mental health problems. Schizophrenia does not follow this pattern, however, which may be attributed to different housing choices for men with schizophrenia. These findings suggest that (1) clinicians providing services to patients in correctional settings need to be trained on how to provide effective health care to individuals living with mood/anxiety disorder; (2) it may be necessary to provide comprehensive integrated (psychological/medical) care services to these inmates; and (3) prisons may not be safe places for persons living with mood/anxiety disorder.

The only enabling factor that was significant is phone calls from family and friends. Thus, social support may be an important factor for encouraging inmates to use health services. Including additional indicators of subjective social support in future studies may help to better understand how extra-prison social bonds influence use of health services behind bars. Enabling factors, in general, are important because they can be modified (unlike predisposing factors). For example, prisons can decrease barriers to maintain extra prison social bonds or provide support for family reunification efforts.

Conceptually, the varying patterns of health service use across prisons suggest that incarceration is a social circumstance that shapes health differentials (Galea & Vlahov, 2002). For example, previous attempts at understanding use of health services in prison focused only on inmate-level predisposing and mediating (enabling) factors even though criminological research has consistently documented that the prison environment is an important determinant of inmate behavior including use of health services as suggested by Anno (1997). This study finds that there is a significant and troubling variation across U.S. prisons in terms of health service use. Moreover, there are both direct and indirect effects of this variation. Wacquant (2000) argues that healthcare in prison may act as a “stabilizing and restorative force,” and black men are more likely to utilize health services which has the potential to improve health for the high proportion of black men cycled through U.S. prisons. In fact, research documents a mortality advantage for black men who are incarcerated (Rosen, Wohl, & Schoenbach, 2011; Wildeman, Carson, Golinelli, Noonan, & Emanuel, 2016). Unfortunately, this study cannot account for the quality of services or differential adherence, and any health benefit afforded in prisons may be outweighed by the long-term health consequences of incarceration (Massoglia, 2008; Massoglia, Pare, Schnittker, & Gagnon, 2014; Porter, 2014; Spaulding et al., 2011). For example, Patterson (Patterson, 2013) analyzed 15 years of administrative data from New York and found a dose-response of time serviced in prison on mortality. Each additional year served in prison produced a 15.6% increase in the odds of death for parolees, which translates to a 2-year decline in life expectancy for each year served.

There are a number of important study limitations to consider. First, this study relies on self-reported health conditions. However, self-report data are an essential and commonly used source of health indicators in research (Stone et al., 1999), and the SISCF is the best data set available to answer the research question because it is the only large, nationally representative survey of inmates available in the United States. It has also been argued that individuals must perceive a need for the utilization of health services (de Boer, Wijker, & de Haes, 1997; Jahangir, Irazola, & Rubinstein, 2012). Related, the data are cross-sectional and do not provide information on onset of health conditions. That is, this study is unable to account for the timing of diagnosis or the severity of symptoms and the timing or quality of care. The data are further limited since there is no way to introduce objective prison-level and state-level controls and must rely on aggregate measures of composition. It is possible that this study has mistakenly assigned variation in inmate medical care usage to prison-level variables rather than differences in custody classification within prisons (Worrall & Morris, 2011). This study does not control for selection effects – neither selection into prison nor selection into need for treatment. Future research should examine selection processes and within prison differences in inmate health behaviors. Finally, future research should give particular attention to incarcerated women because incarcerated women have worse health (Anderson, 2003; Sered & Norton-Hawk, 2008) and lower programming availability (Eliason, Taylor, & Williams, 2004) compared to incarcerated men, and women’s prisons often struggle to meet the healthcare needs of women (Delgado & Humm-Delgado, 2008; Eliason et al., 2004). Therefore, the experience of incarcerated women is shaped by distinct structural processes.

## Conclusion

Even though these data are more than ten years old, this study makes a theoretical contribution by applying the behavioral model of health services use for vulnerable populations to prisons. Future research should apply this model to other types of health service outcomes relevant for inmates such as use of drug treatment services and incorporate objective prison-level factors. It is also possible that predisposing, enabling, and prison-level factors operate differently for women in prison, and for other environments such as federal prisons and local county jails. Nevertheless, this study documents the direct and indirect effects of inmate characteristics and vulnerabilities on use of health services in men’s prisons, as well as the direct effects of the prison enabling and prison-level factors, across multiple types of medical need.

### Notes

^1^A “serious” medical need is defined as “the existence of an injury that a reasonable doctor or patient would find important and worthy of comment or treatment; the presence of a medical condition that significantly affects an individual’s daily activities; or the existence of chronic and substantial pain are examples of indications that a prisoner has a ‘serious’ need for medical treatment” (*McGuckin v. Smith* (974 F.2d 1050 (1992)). See also Ogloff, Roesch, & Hart, 1994 and Wool, 2007. ^2^The final sample size available from BJS is 14,499 persons. In this study, 27 respondents were dropped because they were less than 18 years of age. The number of females dropped is 2927, and the number of individuals who are not serving a sentence is 152.1818 people did not report a medical problem (as defined in this study) and therefore had no information on the outcome of interest because of the skip patterns built into the survey. This leaves a sample of 9191 men who meet the study criteria. 375 of these men have missing information on at least one variable included in the study, which is less than 5%. The listwise deletion leaves the final sample size of 8816 men in 225 prisons. This is a mean of 39 inmates per prison; range 3 to 62. Only three prisons have less than 10 inmates. ^3^BJS recommendations for sample survey weights were followed for all single level analyses. Sampling weights were scaled for multilevel analysis in Stata following Chantala, Blanchette, & Suchindran, 2011. ^4^“Medical care” in this study is used as a general term to differentiate from mental and behavioral health services. ^5^Reported current problems with at least one of the following conditions: heart problems, diabetes, hypertension, cancer, asthma, and kidney problems.
